# Infrared low-level laser therapy enhances proliferation and viability in murine osteoblasts in vitro

**DOI:** 10.1007/s10103-026-04802-x

**Published:** 2026-01-22

**Authors:** Brenda Lizbeth Arroyo Reyes, Luis G. Vázquez-de-Lara-Cisneros, Fabian Galindo Ramírez, Ruben Ramos García, P. Zaca Morán

**Affiliations:** 1 Instituto de Fisiología, Benemérita Universidad Autónoma de Puebla, 72570 Puebla City, Mexico; 2 Facultad de Medicina, Benemérita Universidad Autónoma de Puebla, 72410 Puebla City, Mexico; 3 Coordinación de Óptica, Instituto Nacional de Astrofísica, Óptica y Electrónica, 72840 Puebla City, Mexico; 4 Instituto de Ciencias, Benemérita Universidad Autónoma de Puebla, 72960 Puebla City, Mexico

**Keywords:** LLLT, Osteoblasts, In vitro, Bone regeneration

## Abstract

**Purpose:**

Infrared low-level laser therapy (LLLT) has shown great promise in promoting cell proliferation and viability, making it a valuable tool in regenerative medicine. This study investigated how the interval between sessions shapes the response to 970 nm LLLT in murine osteoblast cultures by delivering three 10 J/cm² sessions separated by 24–48 h and measuring proliferation, reactive oxygen species (ROS), cytotoxicity, and apoptosis, with the goal of informing protocol design for bone regeneration.

**Methods:**

Two osteoblast cultures were used, one control and the other LLL-treated group. The latter consisted of three irradiation sessions (10 J/cm^2^ each) applied at 24, 48, and 96 h.

**Results:**

The experimental results showed a significant increase in cell proliferation after two and three sessions (*p* < 0.05), while ROS levels progressively accumulated, peaking after the third session (*p* < 0.001). Cell viability remained above 90% in both groups during the first 48 h; however, a slight but significant reduction was observed in the LLLT group at 96 h. Apoptosis levels were lower in LLLT-treated cells during early phases (24–48 h), suggesting a transient cytoprotective effect that diminished after the third session. These findings indicate that infrared LLLT promotes cell proliferation without inducing cytotoxicity or programmed cell death.

**Conclusion:**

The results demonstrate that applying three infrared LLLT sessions of 10 J/cm² applied at 24, 48, and 96 h promotes osteoblastic proliferation and viability without inducing cytotoxicity or apoptosis. The proposed protocol, defined by energy dose and irradiation timing, provides a safe and effective strategy for bone tissue engineering.

## Introduction

Photobiomodulation (PBM), historically known as low-level laser therapy (LLLT), has emerged as a non-invasive therapy with broad potential in regenerative medicine [1–3], due to its ability to regulate cellular activity [4], stimulate proliferation, and promote tissue repair without inducing thermal damage [5–7]. The principle of the LLLT is based on the exposure of cell cultures and tissues to low-intensity laser radiation to trigger and enhance interconnected biological mechanisms that contribute to a more efficient reduction of inflammation, pain relief, and improvements in tissue repair and regeneration [8–11]. In particular, this therapy is effective in accelerating processes such as bone remodeling and regeneration [9, 12–14].

Recent studies have shown that LLLT promotes the proliferation and differentiation of osteoblasts, which are responsible for the synthesis and mineralization of the bone matrix during the stages of bone formation and remodeling [15–17]. The modulation of these processes is achieved through the application of low-intensity light that initiates intracellular signaling cascades, which support osteoblastic function and maintain bone tissue integrity [17, 18].

The influence of different LLLT dosages on the viability and cellular activity of osteoblasts, osteocytes, and osteoclasts has been explored extensively [15, 16, 19]. The effects of various LLLT doses on the viability and activity of these bone cells using a 940 nm diode laser were studied by Sungsoo Na et al. [20]. The results showed that a low dose of approximately 1 J/cm² stimulated osteoblast proliferation and bone resorption activity without affecting osteocytes. In contrast, higher doses (5 and 7.5 J/cm²) notably reduced the viability of osteocytes and osteoclasts, and only the highest dose of 7.5 J/cm² harmed osteoblasts. It is worth noting that the experimental design of that study did not include spaced irradiation intervals, which may have limited the optimal metabolism of laser energy and affected cellular proliferation.

Deana et al. have demonstrated that LLLT accelerates alveolar bone healing in animal models by enhancing osteoblastic activity and promoting the synthesis of organized collagen fibers, which contribute to the structural organization required for bone remodeling [21]​. Additionally, other studies have shown that LLLT influences key metabolic pathways, such as the activation of cytochrome c oxidase in the mitochondrial respiratory chain, promoting ATP synthesis and modulating cellular oxidative stress [8]. In the clinical field, LLLT has been evaluated by Ganjeh S. et al. in conditions such as knee osteoarthritis, where improvements were observed in pain control and joint function, particularly when combined with other conservative interventions such as therapeutic exercise [22]​.

Recent studies have explored the effects of PBM on osteoblast cultures. Bomfim et al. [23] evaluated the cumulative effects of PBM using an infrared laser in vitro cultures of human osteoblasts, reporting statistically significant changes in cell viability and proliferation (*p* < 0.05). However, their experimental design differed from the present study in terms of laser wavelength, irradiation intervals, and total delivered dose. Furthermore, other studies have reported proliferative responses to PBM at energy densities around 10 J/cm² in osteoblastic cultures [24, 25] indicating that recovery intervals of 24–48 h support mitochondrial homeostasis and facilitate the reduction of ROS, whereas shorter intervals may lead to overstimulation or oxidative stress [20, 25–27].

In this study, we present the effects of LLLT using a 970 nm infrared laser in vitro cultures of murine osteoblasts. Three sessions of 10 J/cm² were delivered with 24–48 h recovery (total 30 J over 96 h), and proliferation, reactive oxygen species, cytotoxicity, and apoptosis were quantified. The experimental design was based on evidence indicating that incorporating biologically meaningful intervals contributes to the maintenance of cell viability and enhances the proliferative response under controlled conditions, potentially minimizing cumulative oxidative stress and enhancing cellular responsiveness. As this temporal variable remains insufficiently standardized in vitro models, we aimed to address a poorly explored factor that may modulate recovery dynamics between irradiations.

## Materials and methods

### Cell culture

Murine osteoblastic cells used in this study were obtained following the protocol described by Enríquez J. et al. in 2007 [28]. The cultures were maintained in high-glucose, phenol red-free Dulbecco’s Modified Eagle Medium (DMEM), supplemented with 0.28 mM ascorbic acid and 1% of an antibiotic-antimycotic solution (100 U/ml penicillin, 100 µg/ml streptomycin, and 250 ng/ml amphotericin B), and 10% fetal bovine serum (FBS). The incubation conditions were set at 37 °C in a controlled atmosphere with 5% CO₂ and high humidity. These experimental parameters were selected based on previous studies that demonstrated their effectiveness in preserving the viability and functionality of osteoblasts in vitro [29].

The experimental cell cultures were maintained in high-glucose DMEM supplemented with ascorbic acid (0.28 mM) and reduced FBS at 0.2%. Two experimental groups were defined: a control and an LLLT-treated group.

### Experimental setup

**Fig. 1:**
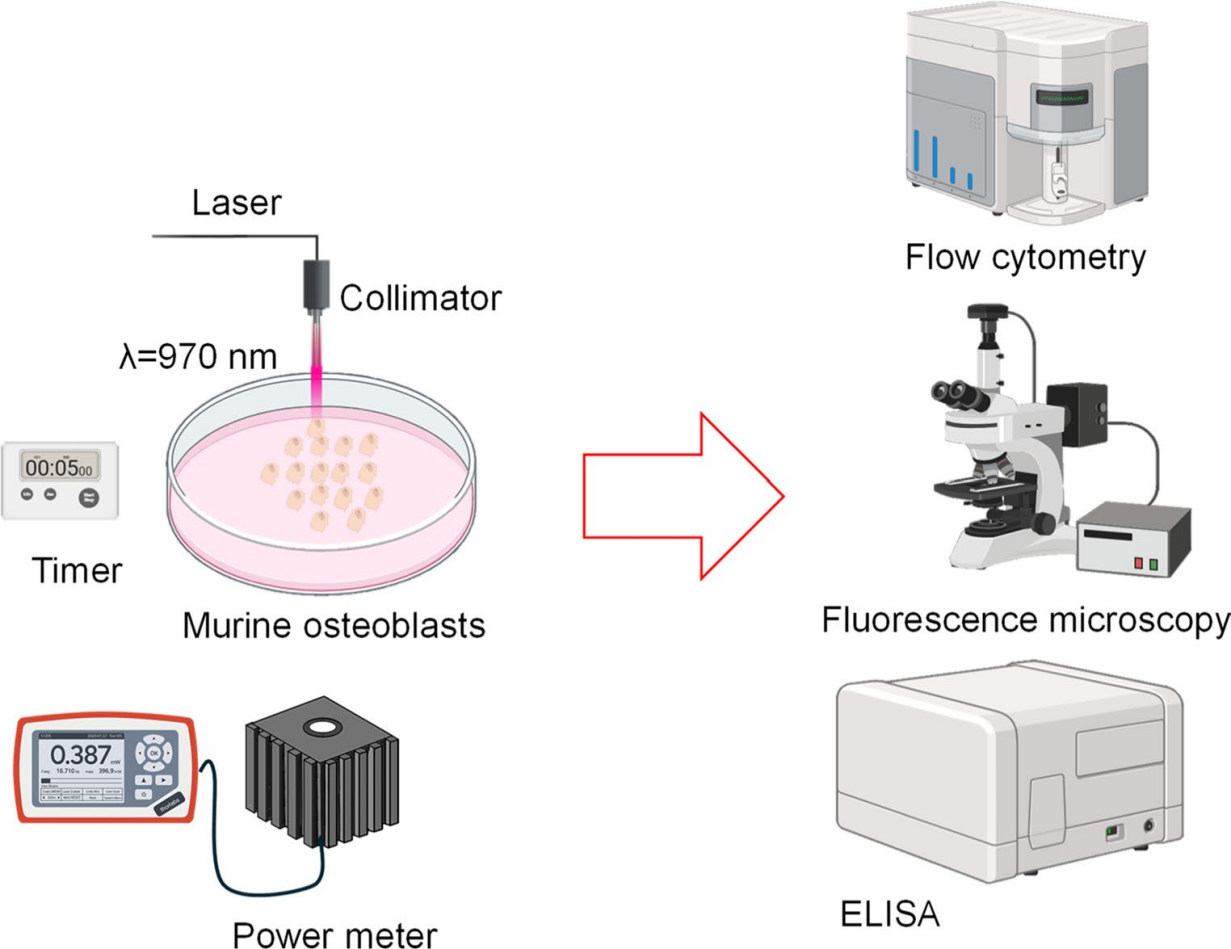
Experimental setup for LLLT at 970 nm on murine osteoblast cultures. Analyses included flow cytometry, fluorescence microscopy, and ELISA

Each treatment session involved continuous irradiation for 300 s, uniformly applied to ensure controlled exposure of the cultures. An optical fiber collimator directed the laser beam onto the cell samples, while a power meter was used to monitor the output and confirm that the delivered dose remained constant across all sessions (Fig. 1).

The group treated with LLLT in vitro cultures of murine osteoblasts was irradiated using a 970 nm infrared laser (model BWF-970-450E/55373, B&W Tek, Inc., USA) with an output power of 7.58 mW. The applied energy density was 10 J/cm² per session over an area of 0.22 cm², with an irradiation time of 300 s per session, resulting in a total accumulated dose of 30 J/cm² by the end of the experimental protocol. Irradiations were performed at 24, 48, and 96 h, following the experimental design established to evaluate the temporal effects of LLLT on osteoblasts.

### Cell proliferation

Cell proliferation was evaluated in 96-well plates by seeding 2,500 cells/mL. Quantification was performed using the WST-1 Cell Proliferation Assay kit (12352200, Roche, Germany), following the manufacturer’s instructions. Measurements were performed by spectrophotometry using the ELISA reader Filter Max F5 (Molecular Devices).

### Reactive oxygen species

Reactive oxygen species (ROS) detection and quantification were performed in 96-well plates seeded with 5,000 cells/mL. The ELISA Reactive Oxygen Species kit (ROS Elisa Kit, EK710415, AFG Scientific), following the manufacturer’s instructions. Absorbance readings were obtained using the ELISA microplate reader FilterMax F5 (Molecular Devices, USA).

### Cytotoxicity characterization

Cytotoxicity evaluation was performed using flow cytometry with the LIVE/DEAD™ viability/cytotoxicity kit (L3224, ThermoFisher), following the manufacturer’s instructions. The assays were carried out in 12-well plates with an initial cell concentration of 10,000 cells/mL. Cell viability was measured by flow cytometry using the CytoFLEX system (Beckman Coulter). Complementary morphological analysis was performed using the ZOE Fluorescent Cell Imager (Bio-Rad, USA).

### Cell apoptosis

Cell apoptosis was evaluated in 12-well plates using an initial seeding density of 10,000 cells/mL. Apoptosis quantification was performed using annexin V-FITC and propidium iodide (PI-ECD) staining (P3566, ThermoFisher Scientific, USA). Flow cytometry analysis was conducted using the CytoFLEX system (Beckman Coulter, USA).

### Statistical analysis

Statistical analysis of the data was performed using a two-way analysis of variance (ANOVA) with Origin 2022 software (OriginLab Corporation, USA). Values of *p* < 0.05 were considered statistically significant. All experiments were performed in triplicate under controlled conditions.

## Results

The results presented in this study describe the response of murine osteoblasts under the influence of LLLT. First, cell proliferation is quantified, which indicates the capacity of the cells to multiply during the treatment. Then, the generation of ROS is analyzed, as these molecules are involved in cellular signaling and metabolism. Cytotoxicity is then evaluated to determine whether laser exposure induces damage or non-programmed cell death. Finally, apoptosis is examined as a regulated mechanism of cell death involved in maintaining cellular balance.

### Cell proliferation

**Fig. 2:**
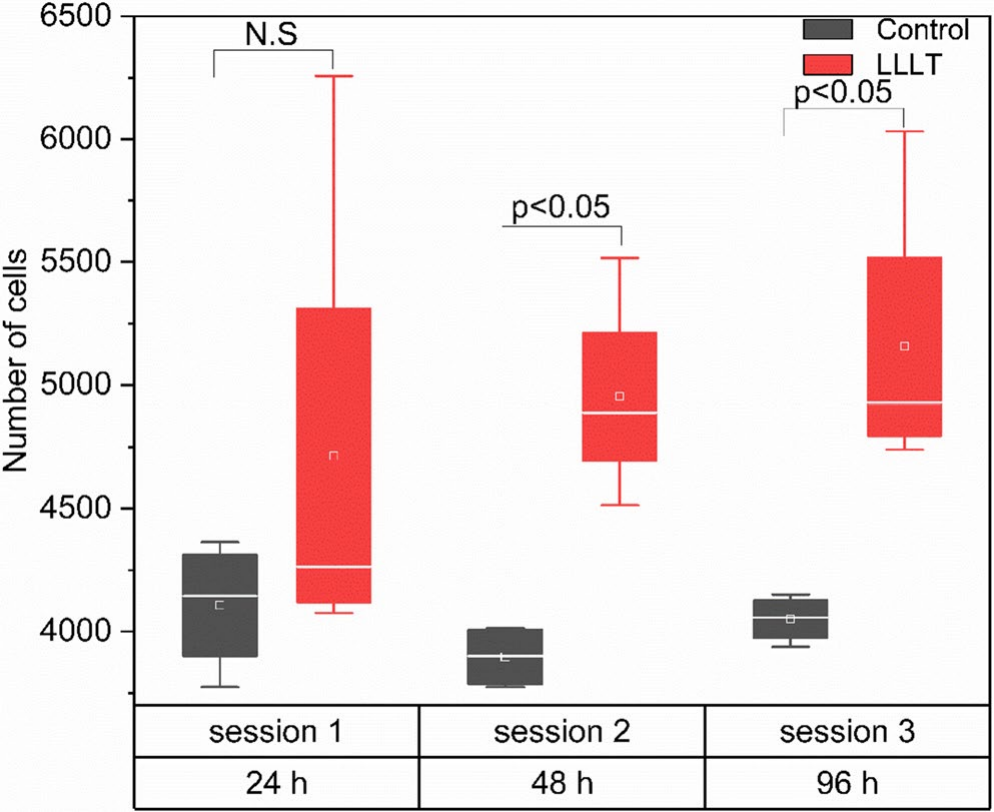
Effect of LLLT on the proliferation of osteoblasts cultured. The number of viable cells is shown at 24, 48, and 96 h after one, two, and three irradiation sessions. Data are presented as box plots comparing the control group (black) and the LLLT-treated group (red). Horizontal lines inside each box indicate the medians

Figure 2 illustrates the number of cells in the control and LLLT-treated groups across the evaluated time points. Osteoblast proliferation was assessed at 24, 48, and 96 h, corresponding to one, two, and three irradiation sessions, respectively. At 24 h, no statistically significant differences were observed between groups (*p* > 0.05), although the irradiated group exhibited greater variability, suggesting a heterogeneous initial response. In contrast, at the second session (48 h), a significant increase in the number of cells was recorded in the LLLT-treated group compared to the control (*p* < 0.05), an effect that persisted after the third session (96 h; *p* < 0.05). These findings indicate that LLLT induces a proliferative effect in murine osteoblasts cultured at low density, with a response dependent on the cumulative number of applied sessions.

### Reactive oxygen species

**Fig. 3:**
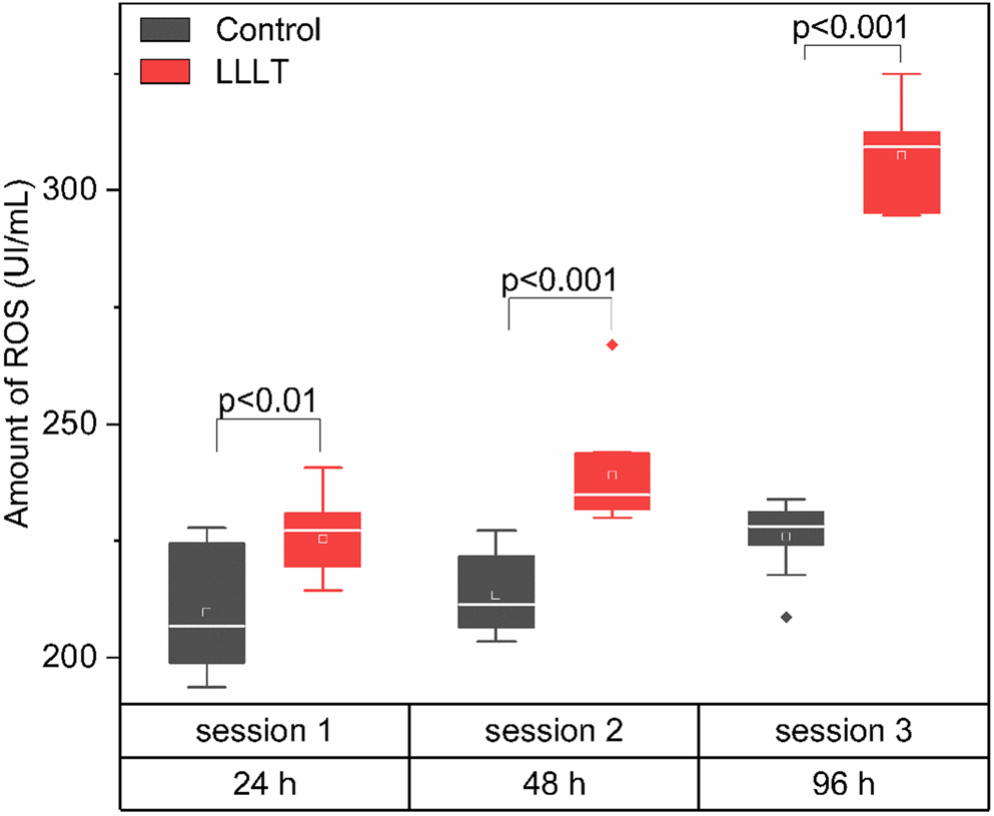
Production of ROS in osteoblasts after treatment with LLLT at different exposure times. ROS levels (UI/mL) were measured in three sessions corresponding to 24, 48, and 96 h post-treatment. The values represent the distribution of the data using box plots for the Control (black) and LLLT-treated (red) groups

Figure 3 shows the ROS in osteoblasts treated with LLLT compared to the control group, evaluated after 1, 2, and 3 therapy sessions. ROS quantification revealed a progressive increase in production levels over time in the group treated with LLLT, compared to the control group.

In session 1 (24 h), ROS levels in the LLLT group were statistically significant in the control group (*p* < 0.01), with median values of approximately ~ 225 UI/mL and ~ 210 UI/mL, respectively. This trend intensified in session 2 (48 h), where the treated group showed a significant increase in ROS production compared to the control (*p* < 0.001), with median values of ~ 240 UI/mL for the LLLT group and ~ 210 UI/mL for the control. In session 3 (96 h), the highest increase in ROS production was observed in the LLLT group, reaching levels close to 310 UI/mL, while the control group remained around ~ 220 UI/mL. The difference between the two groups was highly significant (*p* < 0.001). The observed variability was low within each group, as indicated by the compact distribution of values in the box plots and the presence of a few outliers.

### Cytotoxicity

**Fig. 4:**
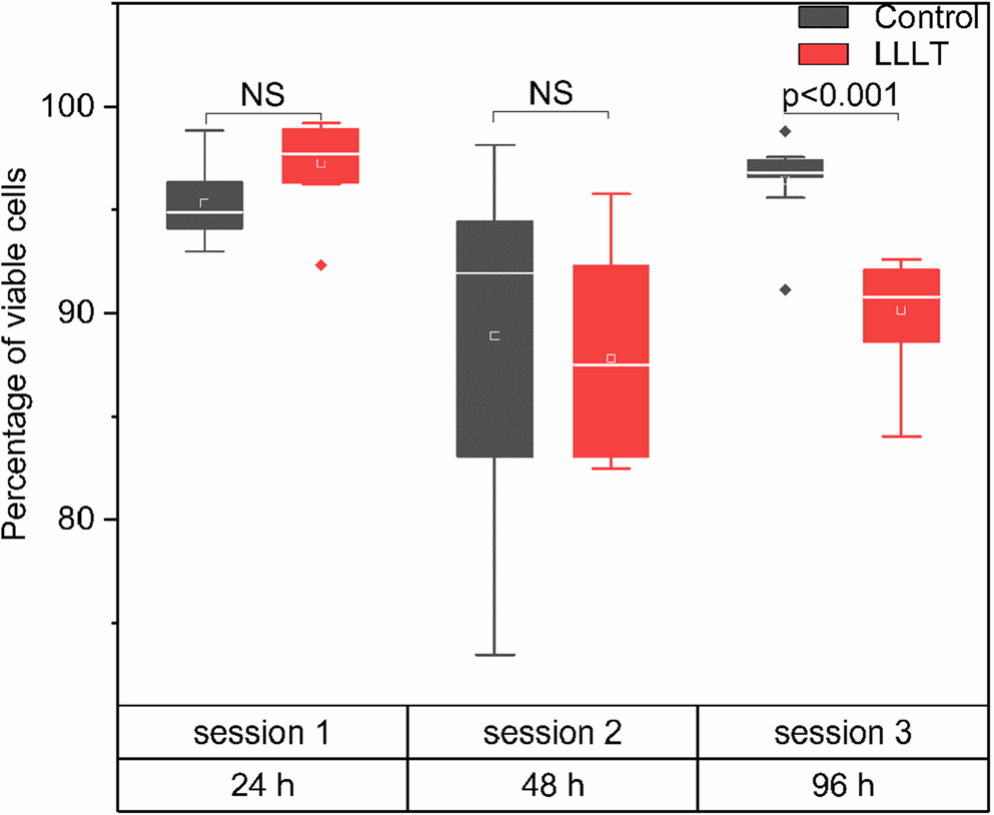
Viability percentage in osteoblast cultures treated with LLLT at different exposure times. Box plots are shown for the Control (black) and LLLT (red) groups across three sessions corresponding to 24, 48, and 96 h

The analysis of cell viability percentage (shown in Fig. 4) revealed overall stability during the first 48 h in both the control group and the LLLT-treated group. In session 1 (24 h), no statistically significant differences (NS) were observed between the two groups with median viability values above 95%, indicating that the initial application of LLLT does not compromise cell viability at this exposure time. Similarly, session 2 (48 h) showed no statistically significant differences (NS) in viability between the groups, although greater data dispersion was observed.

However, in session 3 (96 h), a significant decrease in the percentage of viable cells was observed in the LLLT-treated group compared to the control group (*p* < 0.001). While the control group maintained viability close to 98%, the LLLT group showed a median around 90%, with greater interquartile variability. Notably, cell viability remained above 90% in the LLLT group, confirming that the therapy did not induce appreciable cytotoxicity. The slight reduction in viability at 96 h is likely attributable to increased cell density in the treated wells, leading to contact inhibition and competition for nutrients, rather than a direct toxic effect of LLLT. This effect may trigger cellular self-regulation mechanisms, including the activation of apoptotic processes [12].

**Fig. 5:**
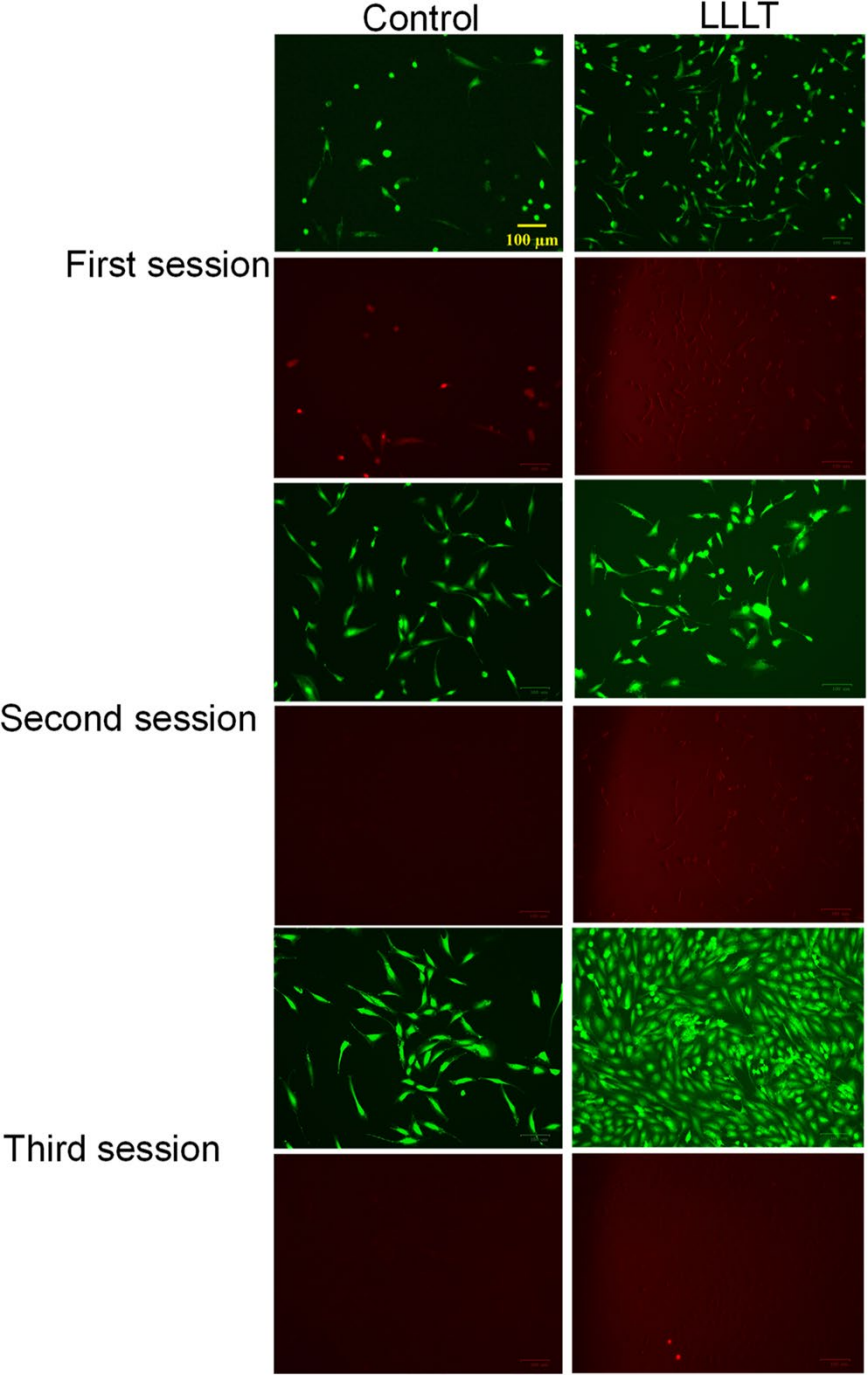
Fluorescence micrographs obtained using the Dead/Live Cell assay to evaluate cell viability under Control and LLLT conditions. Live cells are stained green (calcein AM), and dead cells are stained red (EthD-1). Scale bar: 100 μm

The cytotoxicity results obtained through fluorescence microscopy are shown in Fig. 5. This technique enabled the distinction between live cells, stained with Calcein AM (green), and dead cells, stained with EthD-1 (red), allowing direct comparison between the Control and LLLT groups. In the Control group, osteoblasts maintained a well-defined morphology with consistent green fluorescence across all sessions. In contrast, the LLLT-treated group showed a progressive increase in cell confluence and green fluorescence, consistent with a higher number of viable cells. After 96 h of treatment, cultures exposed to LLLT showed high cell density, green fluorescence, and minimal presence of red-stained dead cells compared to the control group. These results confirm that LLLT had a favorable influence on murine osteoblast proliferation and viability. The minimal presence of dead cells is consistent with a slight increase in cell death, attributable to increased cell density, leading to contact inhibition and competition for nutrients.

### Apoptosis

**Fig. 6:**
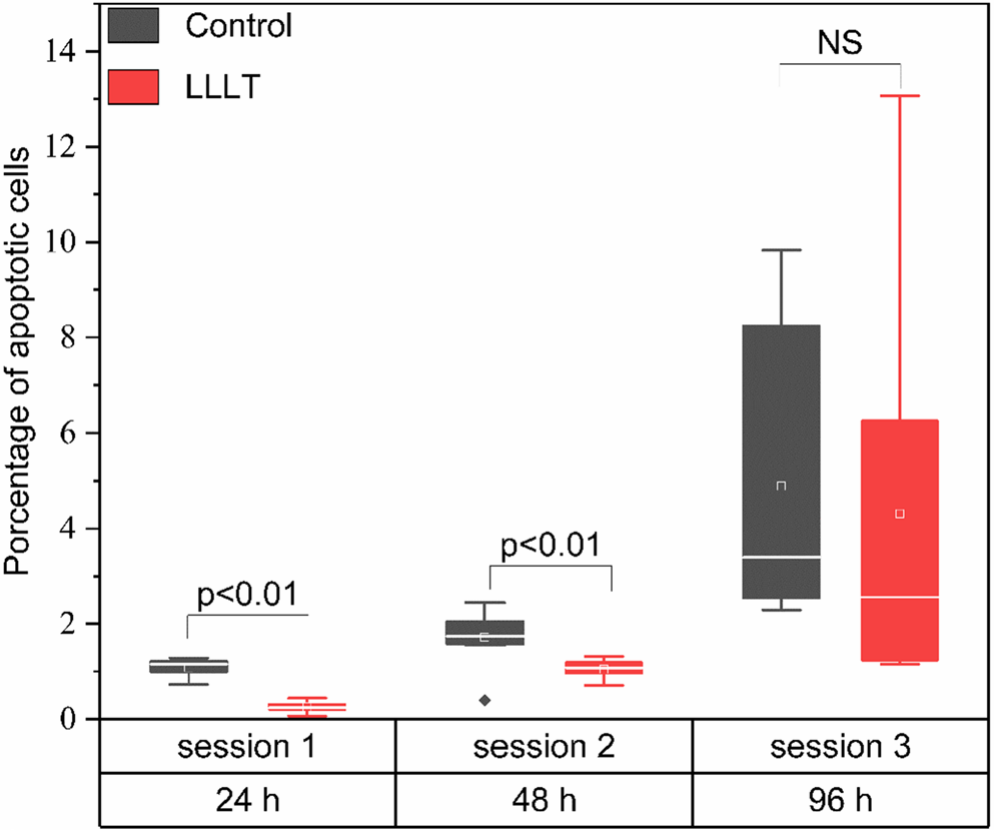
Percentage of apoptotic cells in murine osteoblast cultures treated with LLLT at different exposure times. Boxplots are shown for three sessions (24, 48, and 96 h) comparing the Control (black) and LLLT (red) groups

Figure 6 shows the percentage of apoptosis in cells treated with LLLT compared to the control group. The analysis of apoptotic cell percentages showed time-dependent modulation following LLLT treatment. In session 1 (24 h), the LLLT group showed a significant reduction in apoptotic cells compared to the control (*p* < 0.01), with median values below 1% versus approximately 1.5% in the control. This effect persisted at 48 h, where the LLLT group again exhibited a significantly lower percentage of apoptosis (*p* < 0.01).

In session 3 (96 h), no statistically significant differences were found between the groups (NS); however, a greater dispersion of the data was observed, particularly in the LLLT group, where values ranged up to over 12%. This increase in variability may reflect a heterogeneous cellular response to prolonged irradiation.

## Discussions

In this study, we observed an increase in osteoblast proliferation under LLLT, consistent with Hamblin et al. [30], who showed that infrared-laser PBM promotes cell proliferation through activation of intracellular signaling pathways linked to energy metabolism and osteogenic differentiation. This response is associated with modulation of mitochondrial activity, increased ATP synthesis, and consequent promotion of proliferation without compromising cell viability [12, 15]. Similarly, Bomfim et al. [23] reported increased proliferation altered calcium-signaling gene expression in human osteoblasts following irradiation with an 808 nm GaAlAs laser delivering 37 J/cm^2^ per exposure administered daily for 13 days. By contrast, Huertas et al. [31] studied osteoblast-like MG-63 cells irradiated with a 940-nm pulsed diode laser (fluences 1–5 J/cm^2^) and reported a bell-shaped dose-response: proliferation increased up to ~ 3 J, but then plateaued and, in some conditions, declined at higher fluences. The difference from our findings (no adverse effects at 10 J/cm^2^ with 24–48 h recovery between sessions) reflects differences in dosimetry and timing: Huertas et al. assessed single exposures with outcomes measured 24 h after irradiation, whereas our protocol delivered 10 J/cm^2^ per session across three sessions separated by 24–48 h, allowing recovery between exposures and avoiding overstimulation. Taken together, these observations suggest that both temporal spacing between sessions and dose are critical determinants for maintaining osteoblastic viability and function while promoting proliferation.

ROS levels rose significantly in the LLLT-treated group, suggesting that their production is regulated by the photobiological effects of the therapy. This increase is attributed to the photochemical activation of mitochondrial chromophores, particularly cytochrome c oxidase (COX), recognized as the principal photoacceptor within the 700–1100 nm range [15, 32, 33]. Photon interaction with COX alters the intracellular redox state and, at moderate levels, is consistent with mitohormesis; under this framework, controlled oxidative stress promotes beneficial adaptive cellular responses [34, 35].

Regarding ROS, our results are consistent with those of Chi-Hao Chen et al. [36], who showed that PBM-induced ROS function as signaling molecules within cells, promoting osteoblastic differentiation, cell proliferation, and bone matrix synthesis [15, 27, 37, 38]. The same authors also demonstrated that exogenous antioxidants completely inhibited NF-κB activation triggered by 810 nm irradiation [39, 40], supporting the role of ROS as second messengers in signal-transduction cascades. This pattern aligns with the dual nature of ROS: at moderate levels, they act as beneficial cellular modulators, whereas excessive levels or prolonged exposure lead to cytotoxicity [15, 40]. In this context, our data indicate that a controlled increase in ROS induced by LLLT serves as a relevant mechanism for proliferative signaling in osteoblasts.

In our study, three LLLT sessions were delivered (total accumulated dose 30 J/cm^2^ with 24 and 48 h spacing, which maintained cell viability above 90% during the first 48 h and yielded a value close to 90% at 96 h. Fluorescence microscopy showed a homogeneous osteoblastic morphology with no signs of structural damage. These results differ from those of Na et al. [20], who reported a significant decrease in viability in MC3T3-E1 cells after delivering 7.5 J/cm^2^ with viability assessed at 12 and 24 h post-exposure, suggesting that even moderate doses can induce cytotoxicity. Taken together, the data indicate that both energy density and the interval between sessions are important parameters for designing LLLT protocols aimed at preserving the morphology and viability of murine osteoblasts in vitro.

Table 1 presents a comparative synthesis of in vitro PBM studies on osteoblastic cells, detailing variations in wavelength, fluence, number of exposures, and recovery intervals. Previous investigations employing single or high-dose irradiations frequently reported inconsistent or suppressive effects on proliferation; for example, 12 J/cm² at 980 nm failed to stimulate MC3T3-E1 cells [24]. Agas et al. [24] further demonstrated that 980 nm exposures induced proliferation only at higher energy densities (45 J/cm²), whereas lower doses resulted in neutral or inhibitory responses. In contrast, our 970 nm protocol (10 J/cm², three sessions spaced 24–48 h) maintained cell morphology and viability without signs of cytotoxicity. These results are consistent with those of Berni [26], who reported enhanced osteoblastic proliferation with repeated low-intensity PBM, attributed to mitochondrial regulation and improved ROS regulation.

**Table 1:** Comparative analysis of reported LLLT parameters and their effects on osteoblastic proliferation and viability

Cell model	λ (nm)	Energy density (J/cm²)	No. of sessions/Interval	Main findings	References
MC3T3-E1	940 (LED)	1, 5, 7.5	Single 10-min session (evaluated at 12, 24, and 48 h)	1 J/cm² significantly increased proliferation at 48 h. 5 J/cm² showed no effect. 7.5 J/cm² reduced cell viability.	Na et al. 2018 [20].
MC3T3-E1	830 (Laser)	1, 5, 10	Single session (evaluated at 24 h)	1 J/cm² also significantly increased proliferation at 24 h 5 J/cm² also significantly increased proliferation at 24 h. 10 J/cm² significantly increased proliferation at 24 h.	Renno et al., 2007 [25]
MC3T3-E1	980 (Laser)	12, 27, 45	1, 3, and 5 sessions (1 min per session, weekly sessions for 2 weeks)	12 J/cm² had no effect on proliferation or viability. 27 J/cm² reduced proliferation. 45 J/cm² increased proliferation and activated PI3K/Akt/Bcl-2.	Agas et al., 2021 [24]
MC3T3-E1	660 y 780 (Laser)	90, 150	Single session (exposure of 90 and 150 s)	Neither wavelength (660–780 nm) promoted osteoblastic growth or differentiation at high fluences (90 and 150 J/cm²).	Pacheco et al.,2013 [41]
Murine osteoblasts	970 (Laser)	10	3 sessions (5 min per session)/24–48 h	The protocol with 24–48 h recovery intervals preserved cell morphology and viability without inducing cytotoxicity.	Present work

Taken together, the results of this study show that osteoblast viability and proliferation depend directly on accumulated energy dose, session frequency, and the interval between LLLT sessions. Energy density and exposure time are determining parameters for eliciting beneficial responses, since accumulated doses above 20 J/cm^2^ and prolonged exposures have been associated with cytotoxicity in previous studies [27, 42]. In our protocol (three sessions totaling 30 J/cm^2^, spaced 24 and 48 h), built-in recovery periods prevented overstimulation and maintained both viability and osteoblastic morphology. Accordingly, when dosimetry and spacing are set appropriately, LLLT promotes osteoblast proliferation without compromising structural integrity. It is important to note that this study evaluated a single LLLT protocol with fixed inter-session intervals; therefore, the findings reflect responses observed under these specific experimental conditions. Overall, these results provide an experimental basis for optimizing LLLT protocols for applications in tissue engineering and bone regeneration, including implant osseointegration and the treatment of localized bone defects.

## Conclusions

The results of this study demonstrate that using a 970 nm LLLT protocol of three sessions at 10 J/cm² with 24–48 h spacing, an increase in osteoblast proliferation was observed while maintaining cell viability and preserving morphology in vitro. Across the three sessions, cell density rose together with a moderate increase in ROS, without significant cytotoxicity or excessive apoptosis under these conditions. Proliferation, cytotoxicity, and apoptosis assays revealed a consistently low proportion of programmed cell death, maintaining stability even at high confluence. These results were obtained using a three-session protocol spaced 24–48 h apart, a design that favors osteoblastic proliferation and maintains cellular morphology in vitro. Taken together, these results indicate that dose, together with inter-session timing, are important parameters when planning LLLT protocols for bone-related applications. Overall, the study supports the use of LLLT as a reliable and effective adjuvant for bone regeneration and tissue engineering, including implant osseointegration and the treatment of localized bone defects.

## Data Availability

No datasets were generated or analysed during the current study.
